# Factors Associated with HIV/AIDS Diagnostic Disclosure to HIV Infected Children Receiving HAART: A Multi-Center Study in Addis Ababa, Ethiopia

**DOI:** 10.1371/journal.pone.0017572

**Published:** 2011-03-21

**Authors:** Sibhatu Biadgilign, Amare Deribew, Alemayehu Amberbir, Horacio Ruiseñor Escudero, Kebede Deribe

**Affiliations:** 1 Department of Epidemiology and Biostatistics, College of Public Health and Medical Science, Jimma University, Jimma, Ethiopia; 2 Addis Ababa University, Addis Ababa, Ethiopia; 3 Department of International Health, Bloomberg School of Public Health, Johns Hopkins University, Baltimore, Maryland, United States of America; 4 Department of General Public Health, College of Public Health and Medical Science, Jimma University, Jimma, Ethiopia; Penang Medical College, Malaysia

## Abstract

**Background:**

Diagnostic disclosure of HIV/AIDS to a child is becoming an increasingly common issue in clinical practice. Nevertheless, some parents and health care professionals are reluctant to inform children about their HIV infection status. The objective of this study was to identify the proportion of children who have knowledge of their serostatus and factors associated with disclosure in HIV-infected children receiving HAART in Addis Ababa, Ethiopia.

**Methods:**

A cross-sectional study was conducted in five hospitals in Addis Ababa from February 18, 2008–April 28, 2008. The study populations were parents/caretakers and children living with HIV/AIDS who were receiving Highly Active Antiretroviral Therapy (HAART) in selected hospitals in Addis Ababa. Univariate and multivariate logistic regression analysis were carried out using SPSS 12.0.1 statistical software.

**Results:**

A total of 390 children/caretaker pairs were included in the study. Two hundred forty three children (62.3%) were between 6–9 years of age. HIV/AIDS status was known by 68 (17.4%) children, 93 (29%) caretakers reported knowing the child's serostatus two years prior to our survey, 180 (46.2%) respondents said that the child should be told about his/her HIV/AIDS status when he/she is older than 14 years of age. Children less than 9 years of age and those living with educated caregivers are less likely to know their results than their counterparts. Children referred from hospital's in-patient ward before attending the HIV clinic and private clinic were more likely to know their results than those from community clinic.

**Conclusion:**

The proportion of disclosure of HIV/AIDS diagnosis to HIV-infected children is low. Strengthening referral linkage and health education tailored to educated caregivers are recommended to increase the rate of disclosure.

## Introduction

HIV/AIDS is increasingly affecting the health and welfare of children and undermining hard-won gains in child survival in some highly affected countries [1]. Recent estimates from the Joint United Nations Programme on HIV/AIDS (UNAIDS) suggest that, globally, about 2.5 million children younger than 15 years of age are infected with HIV; 90% of whom live in sub-Saharan Africa [2]. As highly active antiretroviral therapy (HAART) becomes increasingly available in low-resource settings, children affected by this disease are living longer [3], experience a less symptomatic early course of the disease and survive to older ages [4], with improved quality of life [3]. Given this scenario, the question of disclosure of HIV status to infected children is becoming increasingly important. Knowledge of HIV status may affect compliance with antiretroviral therapies and influence children's participation in healthcare decision-making [3]. The American Academy of Pediatrics guidelines on disclosure of HIV illness states that all adolescents should know their HIV status, while disclosure should be considered for school-age children [5]. In Ethiopia, it is recommended that adolescents 14 years of age old and older should know their HIV status [6].

Caregivers and healthcare workers are presented with an array of challenges around disclosure, including deciding on what is in the child's best interest and when, why and how information about his/her HIV status should be shared with him/her [7]. Disclosure of a child's HIV/AIDS is becoming an increasingly common clinical issue. Nevertheless, some parents and health care professionals are reluctant to inform the affected children about it. Data from several sites in other countries indicates that between 25% and 90% of school-age children with HIV infection/AIDS have not been told that they are infected [8]–[10].

Consequently, preparing family members for the emotional impact of disclosure is a major task facing providers. Also, disclosure takes on new significance, both within and outside the family, as sexuality becomes a dominant developmental issue during adolescence [11]. In the context of HAART, disclosure may have an important impact on disease progression and clinical management [12]. However, in one study, interviewed caregivers reported low disclosure rates (9%) [13] and healthcare providers reported low levels of direct involvement in disclosure to HIV-infected children (18%) [14]. Despite the importance of HIV disclosure, there has been limited research addressing pediatric HIV/AIDS disclosure, particularly in sub-Saharan Africa.

## Methods

### Clinical setting and Sample

In Ethiopia, the health system is arranged as a four-tier system, which includes: facility, district, city (region/zone/sub) and federal which should be appropriately linked, equitably distributed and managed in a decentralized, participatory and efficient manner. The Government of Ethiopia launched fee-based antiretroviral treatment in 2003 and free HAART in 2005. As of July 2010, about 97,000 adults and 4,800 children are accessing HAART services in the country. Guidelines from the Ethiopian National Pediatric HIV/AIDS Care and Treatment Guideline recommend HAART initiation in infants <12 months of age, as well as for all infants under 12 months of age with confirmed HIV infection, irrespective of clinical or immunological stage. For children 12 months of age or older, the World Health Organization (WHO) Paediatric Clinical Stage 4 disease (irrespective of CD4), WHO Paediatric Clinical Stage 3 disease (irrespective of CD4), WHO Paediatric Clinical Stage 2 disease and CD4 value at or below threshold. WHO Paediatric Clinical Stage 1 disease and CD4 value at or below threshold, and HIV antibody positive infants <18 months of age where virologic testing is not available to confirm HIV infection should be considered for HAART if they have clinically diagnosed severe HIV disease.

The study was carried out in selected antiretroviral therapy units of 5 tertiary level general hospitals in Addis Ababa, Ethiopia (Black Lion, Saint Peter, Yekatit 12, Zewditu, and ALERT) which serve as the major referral and reference hospitals in Ethiopia. According to the report obtained from registration records, at the time of the study, about 1,624 children were registered for HAART in the selected hospitals.

Of 1624 patients on HAART, 390 (100%) scheduled to present for care or pharmacy pick-ups between February 18 and April 18, 2008 fulfilled the inclusion criteria and were offered to participate. In this study most of the patients/caregivers use bus or taxi to attend the clinics.

The study design was a facility based cross-sectional study. This study was nested as part of a large Multicenter Pediatrics Cross-Sectional study that is published elsewhere [15].

Children who fulfilled the following criteria were included in the study: 1) Receiving continuous antiretroviral therapy for the past 12 weeks before the study in the selected hospitals; and 2) caregivers who had been previously counseled on the importance of drug adherence and on how to recognize common adverse drug reactions associated with antiretroviral drugs.

The study and survey instrument were approved by the Institutional Ethical Review Committee of Jimma University and Research Ethics Committee of Addis Ababa Health Bureau. Official letters of co-operation from the above organization and Federal Ministry of Health (FMOH) were given to respective hospitals. Only caregivers gave written consent for participation in the study. Data were collected by five trained HIV counselors, who were trained on how to interview caregivers with sensitivity, empathy and without expressing judgment. Interviews were carried out privately in a separate room in the Hospital where participants were recruited. In order to ensure participants' confidentiality, no names or personal identifiers were included in the written questionnaires. Identification of an informant was only possible through numerical codes.

### Measurements

The outcome for this study was disclosure of HIV/AIDS serostatus to the participating children. Data was collected by structured questionnaire that had not been previously validated, which was originally developed in English and later translated to Amharic and retranslated back to English by a different person to check for consistency. The content of the questionnaire included: socio-demographic and socio-economic characteristics, medication related factors, health care delivery system related factors, which included access to care, quality of services, and diagnosis related items, referral and treatment, medication administration. Three days intensive training was given for all supervisors and data collectors. Data entry and analyses was carried out using SPSS version 12.0.1 statistical packages. One trained data clerk entered and cleaned the data. Stepwise logistic regression was done to identify factors associated with disclosure. Variables that showed statistical significance below or equal to p≤0.05 where retained for the final model.

## Results

### Socio-demographic and economic characteristics

Out of 390 children ages 1–14 (mean = 8.52, standard deviation [SD] = 2.97] years), 243 (62.3%) children were between 6–9 years of age, 215 (55.1%) were girls and 297 (76.2%) caregivers reported being Ethiopian Orthodox Christian. Of the 390 (100%) caregivers, 176 (45.1%) had primary school education; 174 (44.6%) caregivers were married. Two hundred and seventy seven (71.7%) caregivers mentioned that no one had helped the child financially for their treatment needs, while 6.4% biological fathers and 6.4% local Non-governmental Organizations (NGO's) were reported as being responsible for offering financial support to some of the children. The baseline socio-demographic and economic characteristics are presented in Table 1.

**Table 1:** Disclosure status of children on HAART in Addis Ababa, Ethiopia in 2008, by demographic and social characteristics.

Variable	Disclosure status n (%)		P-value
	Not disclosed	Disclosed	
Age of the child (N = 390)			**0.**001
0–5 years (n = 58)	54(18.8)	4(5.9)	
6–9 years (n = 243)	215(66.8)	28(41.2)	
10–14years (n = 89)	53(16.5)	36(52.9)	
Sex of the child(N = 390)			0.349
Boy(n = 175)	141(43.8)	34(50.0)	
Girl(n = 215)	181(56.2)	34(50.0)	
Religion (N = 390)			0.07
Orthodox(n = 297)	239(74.2)	58(85.3)	
Others 1(n = 93)	83(25.8)	10(14.7)	
Caregiver's educational status (N = 390)			0.001
Unable to read and write(n = 94)	64(19.9)	30(44.1)	
Primary (1–8)(n = 176)	155(48.1)	21(30.9)	
Secondary (9–12)(n = 68)	58(18.0)	10(14.7)	
Diploma and above(n = 52)	45(14.0)	7(10.3)	
Marital status of the caregiver(N = 390)			0.049
Single(n = 38)	30(9.3)	8(11.8)	
Married(n = 174)	150(46.6)	24(35.3)	
Divorced(n = 40)	30(9.3)	10(14.7)	
Widowed(n = 138)	112(34.8)	26(38.2)	
Family income (USD per month) (N = 390)			0.026
<11(n = 277)	220(68.3)	57(83.8)	
11 -<16(n = 50)	43(13.4)	7(10.3)	
≥16 (n = 63)	59(18.3)	4(5.9)	
Relation of child-caregiver(N = 390)			0.162
Mother (n = 62)	54(87.0)	8(16.8)	
Grandmother/father (n = 112)	99(88.4)	13(30.7)	
Uncle/aunt (n = 54)	38(70.4)	16(11.8)	
Others 2(n = 162)	131(80.9)	31(40.7)	
Offering financial aid/support for the child(N = 390)			0.499
No one(n = 277)	231(71.7)	46(67.6)	
Others 3(n = 113)	91(28.3)	22(32.4)	
Preferred age of disclosure by the caregivers (years) (N = 390)			0.09
<12(n = 53)	49(15.2)	4(5.9)	
13(n = 157)	126(39.1)	31(45.6)	
>14(n-180)	147(45.7)	33(48.5)	
Do you know any other children in your community who has HIV?(N = 389)			0.816
Yes(n = 119)	99(30.8)	20(29.4)	
No(n = 270)	222(69.2)	48(70.6)	
Received child care grant(n = 390)			0.003
Yes(n = 85)	61(18.9)	24(35.3)	
No(n = 305)	261(81.1)	44(64.7	

1 *Catholic, Protestant and Muslim.*

2 *by himself/herself, Sister, Brother, Father, Both (mother/father) and Foster parents.*

3 *Father, Local NGO, Uncle, Relatives and Family, Exchange rate 1 USD = 9.6 Ethiopian Birr (ETB).*

### HIV infection, diagnosis, and treatment

Out of the 390 respondents, 210 (53.8%) had someone else living with HIV in their home. One hundred seventy seven of the individuals (84.3%) were taking HAART during the survey period, of which 59 (33.3%) were receiving HAART services in the same facility as the child and 27 (15.25%) had the same day appointment as the child. When asked about the time of the child's diagnosis, 320 (82.1%) caregivers knew it, 114 (29%) caregivers said that they had known about the child's HIV serostatus 2 years prior to the survey. Almost half, 191 (49%) children were referred for HIV screening from the hospital's in-patient ward and 131 (33.6%) from the community clinic.

Nutritional support including Ready-to-use Therapeutic Food (RUTF) provision was provided to 260 (66.7%) children from the hospital. Out of our total sample, 343 (88%) caregivers knew when the child had started HAART. From the 390 (100%) children/caretakers surveyed, 186 (54.4%) had started treatment 2 years before the survey was implemented. Of the children who were taking medication other than ARVs, cotrimoxazole, anti-Tuberculosis medication, as well as multi-vitamins were the most frequently used with 360 (92.3%), 68 (17.8%) and 20 (5%) children taking them, respectively.

### Social Support, Disclosure and Perceived Stigma and Discrimination

Three hundred and twenty-two (82.6%) of the children who participated in the study did not know their HIV serostatus. For none disclosures, 104 (32.3%) caregivers reported that their children were told that they had Tuberculosis (TB) and that their children assumed they were being taken to the health facility for TB appointments. Ninety-four (24.1%) of the caregivers had been attending a support group for caregivers of children with HIV and 89 (94.7%) attended a community organization for social support.

When caregivers were asked about the age at which the child should know about his/her serostatus, 180 (46.2%) respondents said that the child should be told about his/her HIV status when he/she was older than 14 years of age, while 54 (13.8%) pointed out that disclosure should be made at the age of 14. When caregivers were asked about who should have the responsibility of disclosing HIV serostatus to the child, 193 (60%) believed that the doctor should be responsible. A total of 270 (69.2%) respondents reported that they knew other children with HIV in the community (Table 2).

**Table 2:** Patterns of disclosure characteristics of caregivers and children in Addis Ababa, Ethiopia [N = 390], April 2008.

Variable	n (%)
Child know his/her HIV status	
Yes	68(17.4)
No	322(82.6)
People who know child's HIV status	
Father	111(28.5)
Mother	124(31.8)
Sibling	60(15.4)
Grandmother	108(27.7)
Aunt	146(37.4)
Uncle	94(24.1)
Others@	111(28.5)
Who do you think should be the person responsible for disclosure of HIV status? *	
Family (father/mother)	99(30.7)
Health worker (doctor/councilor)	193(60)
Child supporter	30(9.3)

@ Teacher/school, Cousins, Neighbors and Grandfather.

*Total does not add up to 390 caregivers given that 68 were already aware of their HIV status. Some percentages don't add to 100% due to rounding.

A total of 78 (20%) children reported being discriminated by their neighbors. Out of the 78 (20%) children that reported discrimination, 10 (13%) were from the HIV disclosed group and 68 (87%) were from the HIV non-disclosed group. One hundred and ninety-five (50%) of the caregivers reported as children or families affected by HIV/AIDS, including orphans are sometimes verbally mistreated.

### HIV/AIDS disclosure predictors

After controlling for the effects of other variables in the multivariate logistic regression analysis, four characteristics were associated with disclosure of HIV status to children. Comparing children in the 10–14 years age group to children in the 0–5 age group and to those in the 6–9 years of age group, we observed that the last two groups are statistically significant less likely to be informed of their HIV status [(aOR = 0.11; 95% CI = 0.03–0.34 and (aOR = 0.19 ; 95% CI = 0.10–0.37, respectively)]. Perceived awareness of a child of caregiver's illness was also found to be associated with disclosure status. Children who were perceived to know their caregivers health problem were statistically significant more likely to be informed about their HIV status than their counterparts (aOR = 2.20; 95% CI: 1.14–4.28). Educational status of the caregivers was also statistically significant associated with disclosure. Children with caregivers that have education at or above primary level are statistically significant less likely to be informed of their result than those with illiterate caregivers (aOR = 0.28; 95% CI: 0.13–0 .54, aOR = 0.33; 95% CI: 0 .13–0.84 and aOR = 0.32; 95% CI: 0.12–0.86 comparing caregiver with no education vs. primary education, secondary education and diploma and above, respectively). Level of referral for HIV screening was associated with disclosure. Compared to children referred from community clinic, those children referred from hospitals (aOR = 2.87; 95% CI: 1.26–6.51) and private practitioners/NGOs (aOR = 3.88; 95% CI: 1.57–9.58) were more likely to be informed about their HIV test results (Table 3).

**Table 3:** Final Logistic Regression Model for Predictors of Disclosure of HIV/AIDS diagnosis to HIV-infected children in Addis Ababa, Ethiopia, April, 2008.

Variables	Disclosure status		Crude OR** (95%CI)	P-value	Adjusted OR (95%CI)	P-value
	Yes n (%)	No n (%)				
Age of the child (years)				0.001		0.001
0–5	4(5.9)	54(16.8)	0.11(0.03–0.32)		0.11(0.03–0.34)	
6–9	28(41.2)	215(66.8)	0.19(0.10–0.34)		0.19(0.10–0.37)	
10–14	36(52.9)	53(16.5)	1.00		1.00	
Child perceived to know health status of caregiver				0.037		0.019
Yes	24(35.3)	59(18.3)	2.43(1.37–4.30)		2.20(1.14–4.28)	
No	44(64.7)	263(81.7)	1.00		1.00	
Educational level of caregiver				0.004		0.002
Unable to read and write	30(44.1)	64(19.9)	1.00		1.00	
Primary (1–8 grade)	21(30.9)	155(48.1)	0.29(0.15–0.54)		0.28(0.13–0 .54)	
Secondary (8–12 grade)	10(14.7)	58(18.0)	0.37(0.17–0.82)		0.33(0 .13–0.84)	
Diploma and above	7(10.3)	45(14.0)	0.33(0.13–0.82)		0.32(0.12–0.86)	
Place of referral for HIV screening				0.019		0.011
From community clinic	10(14.7)	98(30.4)	1.00		1.00	
Hospital in-patients ward	35(51.5)	155(48.1)	2.20(1.05–4.67)		2.87(1.26–6.51)	
PMTCT programme	1(1.5)	19(5.9)	0.52(0.06–4.27)		0.58(0.07–5.03)	
Private practitioner/NGO*	22(32.4)	50(15.5)	4.31(1.89–9.80)		3.88(1.57–9.58)	

*-Non Governmental Organization.

**OR = Odds Ratio.

## Discussion

In this study only 68 (17.4%) children knew their serostatus. This is lower than the 33% reported in a study conducted in Uganda [16] but comparable with other studies conducted in Europe [17], [18]. Generally, the prevalence of disclosure varies widely across studies and settings, from less than 50% to about 75% of children and youths [19], [20]. The lower prevalence of disclosure in our study might be due to fear of stigma and discrimination by the family members that are not aware or/and caregiver's perceived lack of emotional preparedness of the children and if the child is told he/she will reveal to others leading to stigma and discrimination to the family.

In our study, most caregivers prefer to delay disclosure up to older ages (above 14), this being consistent with previous findings [21], [22]. In addition, it has been documented that parents view children over the age of 12 as emotionally mature for disclosure of HIV status [22]–[26]. In many studies, older children was found to be a determinant factor for the children's' knowledge about their HIV status. Bor *et.al* reported 100% disclosure in children 16 years of age and older [27] and likewise; Cohen *et.al* reported that 95% of children older than 10 years of age were aware of their HIV status in Massachusetts [28]. Similar findings were also documented elsewhere [15], [16]. This could be due to the caregivers' belief that at early age, the child is lacking the emotional and cognitive maturity needed to understand the disease and implications [19], [24], [29], [30]. The perception that adolescence is the optimal period for disclosure may relate to the idea that at this life stage, children are now able to cope with this type of information and address any concerns that they may have as they become sexually active (e.g. HIV transmission) [22]. In our analysis we included children less than 3 years old to explore the disclosure status for all pediatric age groups. Their inclusion might reduce the disclosure rate; however they do not represent a significant proportion of the participants so we do not expect that the relationship is significantly affected due to their inclusion.

The relationship between HIV disclosure and educational level has been documented elsewhere [31], [32]. Wiener *et al.* [30] found that more children who knew their HIV status came from families with a higher socio-economic status and as education is a proxy indicator of higher social economic status. In our study, illiterate caregivers were more likely to disclose the child's HIV status than caregivers with a higher educational level. Again, those caregivers who didn't pay for their child's medication before HAART intake were 62% less likely to disclose the child's serostatus. Similar findings were reported by Wiener *et al.* in which more children who knew their HIV status came from families with a higher socio-economic status [30] but opposite to the study found in Thailand, as more children whose caregivers reported having financial problems knew their diagnosis than those whose care givers did not report to have any financial problems [26]. In the Ethiopian context, affluent families might want to keep their family's status quo by avoiding disclosure.

In practical terms, it is difficult for caregivers to handle the psychological adjustments of their HIV infected children. If the child is aware of the health problem of his/her caretaker, disclosure is more likely to occur. A mother's disclosure of any chronic or life-threatening illness to her child is often accompanied by some level of hesitation and/or anxiety regarding the child's reaction [33], [34]. Similarly, mothers with HIV/AIDS might be particularly worried about their children learning of their illness given the stigma associated with the disease, as well as the methods of transmission [21], [35], [36]. In some cases, caretakers feel relieved of the burden of keeping the secret, and less anxious about medical visits and the possibility of accidental disclosure [9], [37]. Once the caregiver's senses that the child has known the caregivers health problem, it might be easier to disclose the HIV status [38]. According to Murphy *et al.* [39] children's' knowledge of maternal HIV/AIDS status is associated with an increase in child psychosocial adjustment, including enhanced self-esteem among children who know of their mother's HIV infection.

The strength of our study is the large sample size, which represents an important amount of caregivers and their children receiving HAART in Ethiopia in multiple treatment sites, which represent the major HAART reference hospitals in Addis Ababa. Some of the limitations that we identified were the following: first, our sample is limited to urban settings, which might curb any extrapolation of our finding to other settings in Ethiopia. We could not outline whether the differences in disclosure status are associated with cultural factors or other characteristics that were not included in this study. In addition, we acknowledge the possibility of potential selection bias in our study; we investigated only HIV disclosure among people living with HIV/AIDS (PLWHAs) under HAART, but HAART may have a confounding impact on disclosure. Finally, the selection of continuous therapy for 12 weeks and previous counseling on adherence and adverse drug reactions-may bias the results of the study.

In conclusion, the rate of disclosure of pediatric HIV positive status was low in children in Addis Ababa. Given that there is no published research found in the country, this finding will provide evidence regarding pediatric HIV serostatus disclosure. To increase disclosure rate, it is important to target children from higher socioeconomic classes and educated caregivers, children referred from community clinics and younger children. In addition, encouraging disclosure of caregivers' health problems might facilitate disclosure. Intensified information education and communication to de-stigmatize the disease might have far reaching impact. Caregivers and health providers should have a co-responsibility to decide on the proper time to disclose. Finally, as more information is known regarding HIV infection in children and young adults who will become sexually active and who might potentially engage in high risk behavior for HIV infection and other sexually transmitted diseases as well as blood borne diseases (Hepatitis C Virus), we need to be aware that current and future guidelines that consider HIV disclosure need to be flexible, so new information can be included with the ultimate goal of improving the life of children living with HIV and of their caregivers.
